# Clinical trials of tyrosine kinase inhibitors for lung cancer in China: a review

**DOI:** 10.1186/s13045-017-0514-z

**Published:** 2017-08-07

**Authors:** Shan Su, Yi-Long Wu

**Affiliations:** Guangdong Lung Cancer Institute, Guangdong General Hospital and Guangdong Academy of Medical Sciences, 106 Zhongshan Er Road, Guangzhou, 510080 China

**Keywords:** Clinical trials, Lung cancer, China, Tyrosine kinase inhibitors

## Abstract

With the development of evidence-based medicine, clinical trials have become necessary for investigating and validating the efficacy of new treatments. Over the past 10 years, several clinical trials of new anticancer agents have been designed and launched in China; this has greatly promoted the development of novel agents as well as of innovative clinical study designs. However, despite the significant advances made in clinical trials for novel agents, improvements are still required. In this mini-review, we will summarize the ongoing clinical trials of small molecular inhibitors for the treatment of lung cancer in China, aiming specifically to highlight the active involvement of China in these clinical studies. Furthermore, we will discuss the urgent need for improvement of clinical trials and anticancer agent research in China.

## Background

**Fig. 1:**
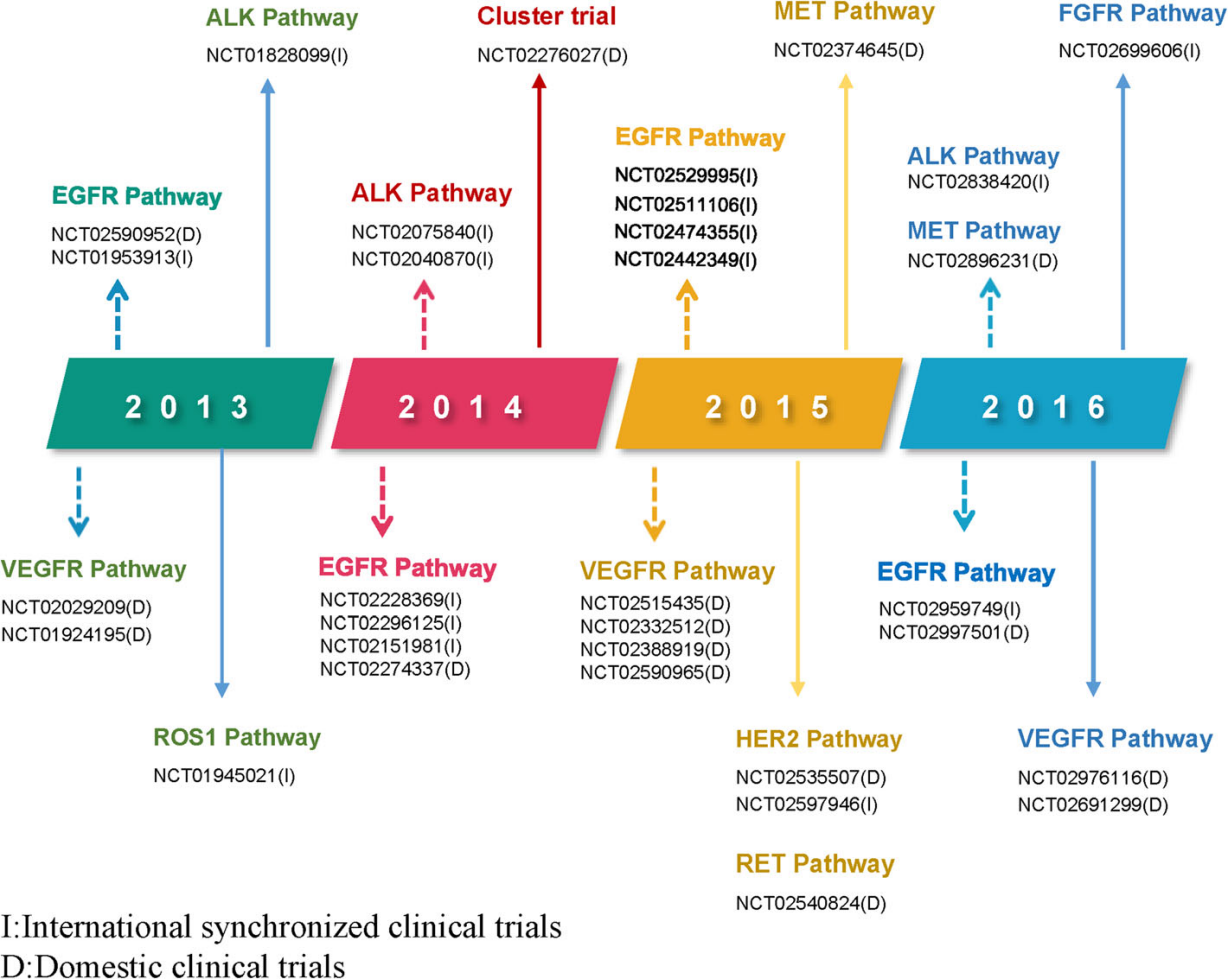
Annual clinical trials of tyrosine kinase inhibitors on lung cancer in China

Significant advances in molecular diagnosis and omics have brought lung cancer treatment into the era of precision medicine. In the past decade, considering the high level of genetic heterogeneity among patients of different ethnicities, many multicenter, international clinical studies of novel anticancer compounds have been conducted in China, particularly on various small molecular inhibitors (Fig. 1). In addition to international synchronized clinical trials, many domestic studies sponsored by Chinese investigators—such as the OPTIMAL and INFORM trials—have also been conducted. Moreover, there has been a quick development of novel antineoplastic agents such as icotinib [a first-generation tyrosine kinase inhibitor (TKI)] in China. These significant advances have changed the clinical treatment of lung cancer in China and have led to a rapid development of clinical trials and, consequently, of new agents. This review will outline the ongoing clinical lung cancer trials, including both domestic and multi-international studies (Table 1), listed on ClinicalTrials.gov and registered with the China Food and Drug Administration (CFDA).

**Table 1:** Ongoing clinical trials of tyrosine kinase inhibitors for lung cancer in China

Agents	Location(s)	Identifier	Phase	PI in China	Indication	Intervention/content	Status in China
Targeting EGFR pathway							
Afatinib	I	NCT01953913	3	Yi-long Wu	Without history of EGFR TKI treatment	Afatinib efficacy in NSCLC with EGFR mutation(s)	Recruiting
Icotinib	D	NCT02714010	3	Li-kun Chen	Without history of EGFR TKI treatment	WBI concurrent with EGFR TKI vs EGFR TKI	Recruiting
Epitinib	D	NCT02590952	1	Yi-long Wu	Without history of EGFR TKI treatment/developed brain lesions during TKI therapy	Pharmacokinetics, safety, and tolerability in solid tumors	Recruiting
Hemay020	D	NCT02467569	1	Hai-ying Wu	Second-line and beyond	Pharmacokinetics, safety, and tolerability in solid tumors	Recruiting
Targeting T790M pathway							
Osimertinib	I	NCT02296125	3	Yi-long Wu	First-line	Osimertinib vs EGFR TKI in advanced NSCLC	Active, not recruiting
Avitinib	D	NCT02330367	1/2	Yi-long Wu	Second-line and beyond	Safety, tolerability, pharmacokinetics, and efficacy in T790M-positive advanced NSCLC	Recruiting
Alflutinib	D	NCT02973763	1	Yuan-kai Shi	Second-line and beyond	Pharmacokinetics, safety, and tolerability in NSCLC after progression on prior EGFR TKI	Not yet recruiting
Targeting HER2 pathway							
Afatinib	I	NCT02597946	2	Cai-cun Zhou	Second-line and beyond	Efficacy in HER2 mutation NSCLC after chemotherapy	Recruiting
Pyrotinib	D	NCT02535507	2	Cai-cun Zhou	Third-line and beyond	Efficacy in HER2 mutation advanced NSCLC	Recruiting
Allitinib	D	CTR20150258	2	Yi-long Wu	Second-line and beyond	Safety and efficacy in uncommon EGFR mutant or HER2 mutation/amplification NSCLC	Recruiting
Targeting ALK pathway							
Alectinib	I	NCT02838420	3	Cai-cun Zhou	First-line	Alectinib vs crizotinib in Asian patients NSCLC	Recruiting
CT-707	D	NCT02695550	1	Yuan-kai Shi	Second-line and beyond	Pharmacokinetics, safety, and tolerability in ALK-positive NSCLC	Recruiting
Ensartinib	D	NCT02959619	1	Li Zhang	Without history of ALK TKI treatment except crizotinib	Pharmacokinetics, safety, and tolerability in ALK-positive NSCLC	Not yet recruiting
SAF-189s	D	CTR20160340	1/2	Yi-long Wu	Second-line and beyond	Safety, tolerability, pharmacokinetics, and efficacy in ALK﻿-﻿positive NSCLC	Recruiting
Targeting c-MET pathway							
INC280	I	NCT02276027	2	Yi-long Wu	Without history of c-MET TKI treatment	INC280 in NSCLC with c-MET gene alteration	Recruiting
Volitinib	D	NCT02374645	1	Jin-ji Yang	Second-line	Volitinib in combination with gefitinib in EGFR-mutated, c-MET-alternated NSCLC after progress on EGFR TKI	Recruiting
Bozitinib	D	NCT02896231	1	Yi-long Wu	Without history of c-MET/HGF inhibitor treatment	Safety, tolerability, and pharmacokinetics in MET-positive advanced NSCLC	Recruiting
BPI-9016M	D	NCT02929290	1	Yuan-kai Shi	Second-line and beyond	Safety, efficacy, and pharmacokinetics in c-MET dysregulated advanced NSCLC	Not yet recruiting
Targeting VEGFR pathway							
Fruquintinib	D	NCT02976116	2	Jian-ying Zhou	First-line	Fruquintinib in combination with gefitinib in non-squamous NSCLC with activating EGFR mutation	Recruiting
		NCT02691299	2	Jian-ying Zhou	Third-line	Placebo-controlled in advanced non-squamous NSCLC	Recruiting
Apatinib	D	NCT02824458	3	Hong-yun Zhao	First-line	Gefitinib in combination with apatinib or placebo in EGFR mutation-positive advanced non-squamous NSCLC	Recruiting
		NCT02332512	3	Li Zhang	Third/fourth-line	Apatinib in advanced non-squamous NSCLC harboring wild-type EGFR	Recruiting
Famitinib	D	NCT02766140	3	Cai-cun Zhou	Second-line	Famitinib plus docetaxel compared to placebo plus docetaxel in advanced or metastatic or recurrent non-squamous NSCLC	Recruiting
Endostar	D	NCT02001168	3	Zuo-liang Pang	Postoperative adjuvant	Comparison of postoperative adjuvant chemotherapy with/without Rh-endostat in phase Ib NSCLC	Active, not recruiting
Anlotinib	D	NCT03059797	2	Ying Cheng	Third-line and beyond	Compare the efficacy and safety of anlotinib vs placebo in SCLC	Recruiting
Targeting RET pathway							
Apatinib	D	NCT02540824	2	Cai-cun Zhou	Second-line and beyond	Efficacy and safety of apatinib as a single agent in RET fusion gene-positive NSCLC	Recruiting
		NCT02780778	2/3	Zhan-yu Pan	Second-line	Apatinib plus docetaxel in advanced non-squamous NSCLC	Recruiting
Targeting ROS-1 pathway							
Crizotinib	I	NCT01945021	2	Jin-ji Yang	First-line or no more than third-line treatment	Safety and efficacy of crizotinib in East Asian patients with ROS1-positive, ALK-negative advanced NSCLC	Active, not recruiting
Apatinib	D	NCT03164694	2	Si-yu Wang	First-line	Apatinib plus chemotherapy vs chemotherapy in ROS1 gene rearrangement advanced NSCLC	Recruiting
Targeting BRAF pathway							
BGB-283	D	CTR20150575	1	Lin-Shen	Second-line and beyond	Pharmacokinetics, safety, and tolerability in advanced solid tumor	Recruiting

*EGFR* epidermal growth factor receptor, *ALK* anaplastic lymphoma kinase, *HER2* human epidermal growth factor receptor-2, *MET* mesenchymal epithelial transition, *VEGFR* vascular endothelial growth factor receptor, *PI* principal investigator, *I* international, *D* domestic

### Agents targeting the epidermal growth factor receptor (EGFR) pathway

EGFR oncogene is the most widely studied driver gene in lung cancer. Currently, the first- and second-generation EGFR TKIs are globally approved for use as standard first-line treatment in patients with EGFR-mutant advanced non-small cell lung cancer (NSCLC). Osimertinib, a third-generation EGFR TKI, received accelerated approval by the US FDA in November 2015 as it was demonstrated to display superiority in terms of the progression-free survival (PFS) and durability of response over platinum plus pemetrexed in EGFR T790M-positive patients after EGFR TKI treatment in a large phase III trial (AURA3, NCT02151981, principal investigator (PI) in China: Yi-long Wu, Guangdong General Hospital) [1, 2]. Based on these promising results, osimertinib was granted accelerated approval by the CFDA in March 2017. An international phase III trial of osimertinib as first-line treatment is now being synchronized in China. Moreover, the fourth-generation EGFR inhibitor EAI045.3, which appears to overcome T790M and C797S resistance, is under preclinical development [3, 4].

Currently, at least six new EGFR TKIs, all independently synthetized in China, are in the early stage of research. Half of these novel agents focus on T790M. In phase I studies, some of these new agents, such as avitinib, have shown excellent responses that are not inferior to those of osimertinib. Accordingly, China has taken a prominent place globally in the research of EGFR TKIs.

#### T790M mutant-selective EGFR TKIs

NCT02296125 (FLAURA, PI: Yi-long Wu, Guangdong General Hospital, China) is a double-blind, phase III study designed to assess the efficacy and safety of osimertinib versus a standard of care EGFR TKI (gefitinib 250 mg or erlotinib 150 mg, once daily) in treatment-naïve patients with locally advanced or metastatic EGFR-mutant NSCLC. Eligible patients were randomized 1:1 to receive osimertinib or a standard of care EGFR TKI. After disease progression, patients in the standard of care group may cross over to receive osimertinib. The primary endpoint is the PFS in each group. The PFS of T790M-positive patients is a key secondary endpoint. This study is being conducted in 31 countries, including at 15 sites in China. The final results are not yet available.

Avitinib, structurally distinct from the pyrimidine-based EGFR inhibitors, is being evaluated in a single-arm phase I/II study (NCT02330367, PI: Yi-long Wu, Guangdong General Hospital, China). The purpose of this clinical trial is to determine the safety, antitumor activity, and recommended phase II dose (RP2D) of avitinib in T790M-positive NSCLC patients. As of July 10, 2016, avitinib has been administered to 136 patients across seven dose cohorts (50, 150, 200, 250, 300, or 350 mg twice daily), and the data from 124 patients are evaluable. The maximum tolerated dose has not been reached. The most common grade 3/4 drug-related adverse events (AEs) were diarrhea (2%), rash (2%), alanine transaminase (ALT) elevation (4%), and aspartate transaminase (AST) elevation (2%). All patients with grade 3/4 AEs recovered after either stopping the treatment or reducing the dose. This study achieved the primary endpoint, with an overall response rate (ORR) of 44% and a disease control rate (DCR) of 85%. In the dose cohorts between 150 and 300 mg twice daily (95 patients), the ORR and DCR were 51% and 89%, respectively. At a dose of 300 mg twice daily (32 patients), the ORR and DCR were 53% and 90%, respectively. Given the safety profile and obvious anti-tumor activity, 300 mg twice daily was selected as the RP2D. The preliminary data will be confirmed in an additional phase III trial (AEGIS-1, NCT03058094) [5].

#### Central nervous system (CNS)-penetrant EGFR TKIs

Patients with EGFR-mutant NSCLC are up to 50% more likely to develop CNS metastasis than those with wild-type EGFR status. However, no small molecular agents have yet been approved for the treatment of CNS metastasis and remain under study. These preclinical agents include osimertinib, which is already on the market and is being tested in patients with the EGFR mutations who have CNS metastases, and another novel agent (AZD3759), which was primarily designed for favorable CNS penetration. China has not been involved in any international clinical trials of CNS-penetrant TKIs. However, dramatic clinical responses were demonstrated in patients with CNS metastases from lung cancer treated with the first-generation EGFR TKI icotinib in a recent phase III trial. In addition, Chinese researchers have synthesized a new compound, named epitinib, which targets brain metastases. A phase I trial of this agent is underway.

In detail, in the phase I dose expansion study (NCT02590952; PI: Yi-long Wu, Guangdong General Hospital, China) of epitinib, EGFR-mutant NSCLC patients with confirmed brain metastases who had either been previously treated with an EGFR TKI or were EGFR TKI treatment-naïve were enrolled. Patients with extra-cranial disease progression while on EGFR TKI treatment were excluded. All subjects will receive oral epitinib 160 mg once daily until intolerance develops or the disease progresses. As of May 31, 2016, the trial has enrolled and treated 27 patients. The most common AEs were skin rash (89%), increased ALT (41%) and AST (37%) levels, hyper-pigmentation (41%), and diarrhea (30%). The most frequent grade 3/4 AEs were elevations in ALT (19%), gamma-glutamyl transferase (11%), AST (7%), hyperbilirubinemia (7%), and skin rash (4%). No grade 5 AEs have occurred to date. Of the 24 evaluable patients, 7 (29%) achieved a partial response (PR), including 1 with an unconfirmed PR. Of these 7 patients, all were EGFR TKI treatment-naïve (7/13, 53.8%). Of the 8 patients with measurable brain metastasis, 40% of treatment-naïve patients had a PR to epitinib [6]. In this study, epitinib displayed clinical efficacy in treating both extra- and intra-cranial lesions. The trial’s final results will be available shortly.

In a phase III study (BRAIN, NCT01724801/CTONG1201 PI: Yi-long Wu, Guangdong General Hospital, China), the EGFR TKI icotinib, developed in China, also demonstrated good efficacy in treating brain metastasis. This randomized study is the first prospective research comparing an EGFR TKI with whole brain irradiation ± chemotherapy in treatment-naïve patients with brain metastases from EGFR-mutant NSCLC. Enrolled patients, randomized 1:1 to the whole brain irradiation or EGFR TKI arm, were administered a total of 30 Gy of radiotherapy (3 Gy/10 fractions) plus 4–6 cycles of concurrent or sequential doublet chemotherapy, or icotinib (125 mg thrice daily), respectively. Crossover to icotinib from the whole brain irradiation arm was permitted. This study has achieved the primary endpoint of intracranial PFS (10.0 vs 4.8 months; hazard ratio, 0.56), as well as the secondary endpoint of median PFS (6.8 vs 3.4 months, *P* = 0.001). The median overall survival (OS) was not significantly different between the two arms (18.0 vs 20.5 months, *P* = 0.734). The intracranial ORR was significantly higher in the icotinib arm than in the whole brain irradiation plus chemotherapy arm (67.1% vs 40.9% *P* = 0.001), as was the ORR (55.0% vs 11.1%; *P* = 0.001), indicating that icotinib is a feasible treatment option for patients with EGFR-mutant NSCLC with brain metastasis [7].

### Agents targeting the anaplastic lymphoma kinase (ALK) pathway

Following the approval of the first ALK inhibitor, crizotinib, the second-generation inhibitors ceritinib and alectinib are now approved for use in the US, the EU, Korea, and Japan for the treatment of ALK-positive patients who have shown disease progression on or are intolerant to crizotinib [8]. In phase III trials (ASCEND-4, NCT01828099; J-ALEX, NCT02075840) on first-line treatment with ceritinib or alectinib, both groups of patients showed a significant PFS benefit compared with patients receiving chemotherapy or crizotinib. Furthermore, brigatinib, a potent dual inhibitor of ALK and EGFR with potential CNS penetration, was granted accelerated approval for patients with metastatic ALK-positive NSCLC who are resistant or intolerant to crizotinib by the FDA on April 28, 2017. The approval was based on findings from a phase II trial (ALTA NCT02094573), in which the confirmed ORR for brigatinib was 53% and the median PFS was 13.8 months. Moreover, the third-generation inhibitor lorlatinib, which was developed as a more potent and CNS-penetrant ALK/ROS1 inhibitor, was also granted a breakthrough therapy designation for use in patients with ALK-positive metastatic NSCLC who have previously received one or more ALK inhibitors in April 2017 [9]. A fourth-generation ALK inhibitor, TPX-0005, was already under investigation in a phase I/II study (NCT03093116).

However, contrary to the rapid development of ALK inhibitors internationally, crizotinib is currently the only agent approved by the CFDA. At present, the only phase III study of ceritinib in China has been completed and no clinical trials of brigatinib or lorlatinib have been designed. In fact, only front-line studies of alectinib are being conducted. Although there are few international clinical trials of ALK inhibitors in China, second-generation ALK inhibitors (such as ensartinib, SAF-189s, CT-707, and PLB1003) have been developed by Chinese investigators; most are still in the early stage of investigation but have shown more potent activity compared to crizotinib.

Among others, ensartinib is one second-generation ALK inhibitor developed in China. In preclinical models, ensartinib demonstrated better anticancer efficacy than crizotinib. It was also found to be active in patients with resistance to first-generation TKIs. It is currently being tested in a phase I/II study (CTR20160340 PI: Yi-long Wu, Guangdong General Hospital, China) to determine its safety, tolerability, and RP2D. In the dose escalation group, patients with ALK-positive advanced solid tumors will be treated with ensartinib 10 mg once daily. In the dose expansion group, patients with ALK-positive advanced NSCLC will be treated with ensartinib 50 mg once daily. All enrolled subjects will receive treatment for 21 consecutive days. The primary endpoints in phase I are the dose-limiting toxicity and ORR. The primary endpoints in phase II are the ORR, AEs, serious AEs, and treatment-emergent AEs.

NCT02838420 is a randomized phase III study (PI: Cai-cun Zhou, Shanghai Pulmonary Hospital, China) that aims to confirm the PFS benefit for patients treated with alectinib in the J-ALEX study (NCT02075840 PI in China: Cai-cun Zhou, Shanghai Pulmonary Hospital, China). This study plans to recruit 183 treatment-naïve Asian patients with ALK positivity as determined by immunohistochemistry staining (D5F3, Ventana). Enrolled participants will be randomized 2:1 to receive either alectinib or crizotinib, until disease progression, unacceptable toxicity, withdrawal of consent, or death. The primary endpoint is the PFS. The secondary endpoints are the percentage of participants with complete or partial responses, and the time to progression to CNS disease as determined by the immune-related response criteria (IRR) set out in version 1.1 of the Response Evaluation Criteria in Solid Tumors.

### Agents targeting the mesenchymal–epithelial transition (MET)–hepatocyte growth factor (HGF) pathway

To date, no agents targeting MET activation in lung cancer have been approved for use. Most novel agents targeting MET, such as MET TKIs and monoclonal antibodies (e.g., crizotinib, cabozantinib, capmatinib, and onartuzumab), are in the early stages of research. In an expansion cohort phase I study (PROFILE 1001, NCT00585195), crizotinib, a multi-kinase inhibitor, showed encouraging ORRs of 44% (8/18) in patients with MET exon 14-altered NSCLC and 50% (3/6) in patients with MET amplification (MET/CEP7 ratio ≥5) NSCLC. When combined with gefitinib, capmatinib (INC280), another MET-selective TKI, also demonstrated a promising ORR of 50% in patients with a MET gene copy number >5 in a phase II study (NCT01610336, PI: Yi-long Wu, Guangdong General Hospital, China). In contrast, tivantinib, a selective MET inhibitor, and onartuzumab, an anti-MET antibody, did not show any OS advantage in several phase III trials.

While a number of new agents targeting MET-active tumors have emerged globally, the only research being conducted in China is on capmatinib. Nonetheless, Chinese researchers are active in ongoing agent development efforts. Several novel MET inhibitors (such as bozitinib, volitinib, and BPI-9016M) have been synthesized and evaluated in early studies. Here, we will discuss the clinical trials of capmatinib and volitinib being conducted in China.

NCT01610336 (PI in China: Yi-long Wu, Guangdong General Hospital, China) is a phase Ib/II study that aims to assess the safety and efficacy of escalating doses of capmatinib plus gefitinib in patients with c-MET-positive, EGFR-mutant NSCLC. Eligible patients were centrally assessed as being c-MET-positive (IHC 3+ or 2+ and gene copy number >5). All participants received the RP2D of capmatinib (400 mg twice daily) + gefitinib (250 mg once daily). As of September 28, 2015, there were 83 patients recruited in the expansion part of the trial. The updated results of the phase II study documented an ORR of 18% in 12/65 evaluable patients and 50% in the MET gene copy number >5 group. The most common all-cause AEs were hypoalbuminemia (29%), peripheral edema (27%), and decreased appetite (23%). The most common grade 3/4 AE (regardless of causality) was an increased amylase level (7%) [10]. This clinical trial has been completed, and the final results will be available in the near future.

Volitinib, a small molecular inhibitor with potent efficacy against c-MET-positive (c-MET overexpression: IHC 3+ or 2+ and gene copy number >5) tumors in preclinical models [11], is being tested in a multicenter phase Ib study (NCT02374645, PI: Yi-long Wu, Guangdong General Hospital, China). This study plans to register 53 patients with EGFR-mutant, c-MET-positive NSCLC who experienced disease progression on previous EGFR TKI treatment. In the safe run-in and expansion cohorts, eligible patients will receive gefitinib plus volitinib 600 or 800 mg once daily, respectively. The primary endpoints are the number of AEs and serious AEs. The secondary endpoints are to establish the pharmacokinetic profile of volitinib, the PFS, and the DCR. The study recruitment was completed in April 2017.

### Agents targeting the vascular endothelial growth factor receptor (VEGFR) pathway

Although many VEGFR inhibitors and antibodies have been approved for use as anti-tumor therapy, only bevacizumab and ramucirumab have been approved for the treatment of lung cancer by the US FDA in 2004 and 2014 respectively. In China, bevacizumab and apatinib are the only two agents approved by the CFDA for lung cancer treatment. There are clear differences in the drugs targeting the VEGFR pathway between those developed in China compared to in other countries. First, most international agents are monoclonal antibodies, such as bevacizumab, whereas Chinese researchers have focused on small molecular multi-targeted inhibitors (such as anlotinib, apatinib, and fruquintinib), which have shown less toxicity but significant activity in clinical trials. Second, in most international trials, agents against VEGFR are usually combined with chemotherapy, using PFS as the primary endpoint. In contrast, Chinese researchers are more inclined to administer single agents in placebo-controlled studies following multiple lines of treatment, using OS as the primary endpoint. Although anti-angiogenesis agents are recommended as first-line therapy, how to change the clinical practice when checkpoint inhibitors become a standard treatment will pose a challenge in the near future. Here, we introduce three new VEGFR TKIs developed in China.

Anlotinib is a novel multi-targeted inhibitor targeting the VEGFR2/3, fibroblast growth factor receptors 1–4 (FGFR1–4), platelet-derived growth factor receptor-α/β, c-Kit, and RET pathways [12]. In a placebo-controlled phase II trial (ALTER0302, NCT01924195), anlotinib showed benefits in PFS (4.8 vs 1.2 months) and OS (9.3 vs 6.3 months) as third-line treatment in patients with advanced NSCLC. A randomized phase II/III trial (ALTER0303, NCT02388919, PI: Bao-hui Han, Chest Hospital affiliated with Shanghai Jiaotong University, China) was conducted to further confirm the encouraging result of anlotinib in the ALTER0302 study [13]. Four hundred thirty-seven patients with sensitive EGFR or ALK mutations, who must have received and have had intolerance to previous targeted therapies, were enrolled in this study. They were randomized 2:1 to receive either anlotinib hydrochloride (*n* = 294, 12 mg) or a placebo (*n* = 143) once daily from days 1 to 14 of a 21-day cycle until disease progression. If the patients did not tolerate the AEs, their dosage was lowered. This study has achieved the primary endpoint of OS (9.6 vs 6.3 months; *P* = 0.0018) as well as the secondary endpoint of median PFS (5.4 vs 1.4 months, *P* < 0.0001) and ORR (9.2% vs 0.7%, *P* < 0.0001).

Fruquintinib, a highly potent and selective VEGFR 1/2/3 inhibitor, showed an acceptable safety profile and significant efficacy in a phase I study [14]. It is currently being evaluated in a randomized phase III study (FALUCA, NCT02691299, PI: Shun Lu, Shanghai Chest Hospital, China) that aims to compare fruquintinib with a placebo in NSCLC patients who either failed two lines of systemic chemotherapy or experienced intolerable toxicities. All enrolled patients will be randomized to the fruquintinib or placebo group in 4-week cycles; patients will receive fruquintinib (5 mg) or placebo daily with best supportive care for three consecutive weeks, followed by 1 week off. Tumor assessment will be performed every 4 weeks during the first 2 cycles and every 8 weeks following the third cycle, until disease progression or death. Their subsequent anti-neoplastic treatment and survival status will be followed up after disease progression. The primary endpoint is the OS, while the secondary endpoints are the ORR, PFS, and DCR.

In 2014, the CFDA approved the use of apatinib, another novel selective VEGFR TKI, for treatment of gastric cancer [15, 16]. Apatinib is now being investigated in NSCLC patients in a double-blind, placebo-controlled phase III trial (ANSWER, NCT02332512, PI: Cai-cun Zhou, Shanghai Pulmonary Hospital, China) as third/fourth-line treatment. This study plans to enroll 40 non-squamous NSCLC patients with wild-type EGFR. All patients will be randomized to receive oral apatinib (750 mg once daily) or a placebo until disease progression. The primary endpoint is the OS. The secondary endpoints are the PFS and ORR.

### Agents targeting the fibroblast growth factor receptor (FGFR) pathway

The first agents to be studied as FGFR inhibitors were multi-kinase anti-angiogenic drugs that were initially developed to target VEGFR, including brevanib, cediranib, dovitinib, lucitanib, and nintedanib. Clinical trials of second-generation selective FGFR inhibitors (such as AZD4547, BGJ398, LY2874455, JNJ-42756493, and FP-1039) have started; most are still in phase I/II [17]. At present, there are no FGFR inhibitors or antibodies approved for use in lung cancer as their clinical efficacy is modest at best. Although FGFR-positive status are relatively rare, Chinese researchers are involved in studies of several FGFR inhibitors, such as BGJ398 and FGF40. Moreover, AL3810, a novel agent developed in China, is being tested in advanced solid tumors in a phase I study.

BGJ398, a selective FGFR1–3 TKI, showed efficacy and a tolerable safety profile in a phase I study (NCT01004224) [18, 19]. It is being tested in another phase I single-arm study (NCT01697605) that aims to evaluate its safety and tolerability to determine the maximum tolerated dose and/or recommended dose. This study is being conducted in Japan and China recruiting patients with advanced solid tumors harboring FGFR alterations. All eligible participants will receive oral BGJ398 once or twice daily until unacceptable toxicity or progressive disease develops. The preliminary endpoints are the dose-limiting toxicity, maximum tolerated dose, and recommended dose. The secondary endpoints are serious AEs, the ORR, and PFS.

Erdafitinib is an orally administered pan-FGFR TKI. In the first human study (NCT01703481), erdafitinib demonstrated pharmacodynamic biomarker activity and safety [20]. Furthermore, a phase II study (NCT02699606) is currently being conducted to determine the efficacy of erdafitinib in Asian patients with advanced NSCLC, urothelial cancer, esophageal cancer, or cholangiocarcinoma. In this single-arm study, participants must meet the molecular eligibility criteria of FGFR alteration and will receive erdafitinib 8 mg once daily with the option to titrate up to 9 mg in 28-day cycles. The primary endpoint is the ORR. The secondary endpoints are the PFS and DCR.

### Agents targeting the human epidermal growth factor receptor 2 (HER2) pathway

Many agents targeting the HER2 pathway (such as herceptin, pertuzumab, and lapatinib) are approved for use in breast cancer; most have displayed a discouraging level of activity in lung cancer. Recently, afatinib, a multitargeted inhibitor, has shown preliminary efficacy in a fraction of patients with HER2-positive lung cancers. Therefore, it is being studied further in a phase II trial in China and Malaysia.

In addition, several small molecular multi-kinase inhibitors developed in China (including dacomitinib, pyrotinib, and allitinib) have been reported to have initial activity in patients with HER2-positive lung cancers. These agents are also undergoing clinical investigations.

NCT02597946 (PI: Cai-cun Zhou, Shanghai Pulmonary Hospital, China) is a single-arm phase II study designed to investigate the efficacy and safety of afatinib in patients with advanced NSCLC with HER2 mutations who were previously treated with one or two chemotherapy regimens. This study comprises two parts (part A and part B) in which 40 patients will be enrolled. All patients will be recruited in part A to receive continuous once-daily treatment with afatinib at a starting dose of 40 mg. Patients who achieved >12 weeks of clinical benefit before disease progression in part A will continue to part B to receive afatinib 40 mg once daily plus paclitaxel 80 mg/m^2^ weekly. The primary endpoint in part A is the ORR; the secondary endpoints are the OS, DCR, and PFS, the time to progression, and the duration of response in part A and the OS in part B [21].

Allitinib is an irreversible inhibitor of EGFR and ErbB2, which was developed in China. The safety and efficacy of allitinib are currently being evaluated in a phase II study (CTR20150258; PI: Yi-long Wu, Guangdong General Hospital, China) on patients with advanced HER2-positive NSCLC for whom previous chemotherapy failed. This single-arm trial plans to recruit 40 patients who will receive allitinib 600 mg once daily until disease progression. The primary endpoint is the ORR; the secondary endpoints are the PFS and OS.

### Agents targeting the ROS-1 pathway

In March 2016, the FDA granted crizotinib the designation of a breakthrough therapy for the treatment for patients with ROS1-positive NSCLC, based on the encouraging results of a phase I expansion cohort study (PROFILE1001: NCT00585195). Several other multi-kinase inhibitors (including ceritinib, lorlatinib, and cabozantinib) have also shown clinical efficacy in patients with ROS1-positive NSCLC in early-stage studies. Confirmatory phase II studies of these inhibitors are underway globally (NCT02183870, NCT01945021, NCT01964157, NCT02927340, NCT01970865); however, only crizotinib is being tested in Chinese patients with ROS1-positive NSCLC.

NCT01945021 is the first study of crizotinib in Asian (Chinese, Japanese, Korean, and Taiwanese) patients with ROS1-positive NSCLC. This phase II study has enrolled 129 patients with advanced NSCLC confirmed by reverse transcriptase-polymerase chain reaction(RT-PCR) to be ROS1-positive; it is the largest such study to date. At the data cutoff date (6 months after the last patient was recruited), there were 127 patients enrolled in the analysis, and 63% of the patients were being investigated. This study met the primary endpoint, providing an ORR (by immune-related response) of 69% (95% confidence interval, 60.5–77.2). The median duration of crizotinib treatment was 7.8 months. The median PFS duration was 13.4 months (95% confidence interval, 10.3 to not achieved), and the median OS has not yet been reached. The most frequently reported all-cause AEs (any grade) were elevated transaminase levels (58%), visual disorders (47%), diarrhea (46%), and nausea (44%) [22].

### Agents targeting the RET pathway

At present, no RET-selective inhibitor has been developed. Patients with advanced RET-rearranged NSCLC have shown clinical responses to treatment with multi-targeted inhibitors such as cabozantinib and vandetanib (drugs approved for use in advanced thyroid medullary carcinoma) in several early studies; the safety profile of these drugs also appears manageable [23, 24]. Other multi-kinase inhibitors (lenvatinib, alectinib, and ponatinib) have also shown efficacy in preclinical models and early-stage trials and are being tested in several phase II studies (NCT01877083, NCT02314481, NCT01813734) [25–27]. However, no trials have been conducted in China.

Apatinib, a multi-kinase inhibitor targeting VEGFR and RET rearrangement is being tested in a single-arm phase II study (NCT02540824, PI: Cai-cun Zhou, Shanghai Pulmonary Hospital, China) that aims to determine the efficacy and safety in RET-fusion gene-positive NSCLC patients for whom previous treatment failed. Enrolled patients will be treated with apatinib 750 mg once daily until disease progression. The primary endpoint is the ORR. The secondary endpoints are the OS, PFS, and quality of life. The results are pending.

### Agents targeting the BRAF V600E pathway

Dabrafenib is a BRAF inhibitor approved to treat patients who express the BRAFV600E mutation, while trametinib, a MEK inhibitor, is approved to treat patients with either the BRAFV600E or BRAFV600K mutation. Both agents act on the RAS kinase pathway. On June 22, 2017, the US FDA granted regular approvals to dabrafenib and trametinib administered in combination for patients with metastatic NSCLC with BRAF V600E mutation. The approval was based on a three-cohort, non-randomized, non-comparative study in patients with BRAF V600E mutation-positive metastatic NSCLC (NCT01336634). In the combination group, the ORRs for the previously treated cohort and treatment-naïve cohort were 63% and 61%, respectively, while the ORR for patients who received single-agent dabrafenib was only 27%. At present, both these drugs are being tested in BRAF V600 positive-mutation melanoma in China.

Although no BRAF inhibitor has yet been granted approval for patients with BRAF V600E-mutant NSCLC by the CFDA, a second-generation BRAF inhibitor named BGB-283 developed in China has displayed potent inhibition in preclinical studies on BRAFV600E and EGFR mutation/amplification. At present, it is being tested in a phase I study enrolling subjects with BRAF, NRAS, or KRAS mutation-positive solid tumors (NCT02610361).

### Innovative clinical trial designs

Over the past decade, Chinese researchers have not only devoted their efforts to develop new anticancer agents (such as icotinib, avitinib, and apatinib) but have also focused on innovative clinical trial designs.

NCT02330367, a dose escalation and expansion phase I/II study, aims to determine the safety profile and RP2D of avitinib in EGFR T790M-positive patients. Most previous clinical trials have determined the RP2D by using only the maximum tolerated dose; however, it is more reasonable to decide the RP2D by establishing the pharmacokinetics, efficacy, and safety profile of an agent. This innovative design has established a new pattern for improvement of efficacy in biomarker-driven clinical trials, allowing a wider effective dose range and less toxicity. Benefitting from this, in 2016, researchers applied for approval by the CFDA to conduct phase II/III clinical trials of avitinib; this is the fastest application of a novel agent in China.

Another innovative clinical research strategy is used in the umbrella trial named “cluster” (NCT02276027, PI Yi-long Wu, Guangdong General Hospital, China). This is the first multiple biomarker-driven clinical research study to be conducted in Asia. The enrolled patients were assigned to groups receiving different agents according to their gene profiles. Five agents targeting different molecular abnormalities are being studied: INC280 for MET-positive patients; LDK378 for ALK or ROS1 rearrangement patients; BYL719 for patients with PIK3CA mutation/amplification; MEK162 for patients with KRAS, NRAS, or BRAF mutations; and allitinib for patients with HER2 or HER4 mutations. Each agent arm will be analyzed independently. If the response rate of one agent reaches 40%, a phase II trial will be conducted. Owing to this multiple-arm parallel assignment, the overall screen failure rate will be reduced, and more patient biomarkers can be matched with agents. Accordingly, this study is a significant milestone in the era of precision medicine in China.

Finally, next-generation sequencing and liquid biopsy are playing emerging roles in innovative clinical trials. One representative trial is the benefit trial (NCT02282267, PIs: Jie Wang, Beijing Cancer Hospital, and Yi-long Wu, Guangdong General Hospital, China). This is the first prospective, multi-center study to validate the efficacy of targeted therapy determined by plasma cell-free DNA (cfDNA). At baseline, all participants underwent EGFR gene profiling performed by droplet digital PCR using plasma samples. For the duration of the treatment period, blood samples were collected every 2 months until disease progression occurs. The primary endpoint is the ORR, and the secondary endpoints are the PFS, DCR, and detection consistency using droplet digital PCR for plasma or the amplification-refractory mutation system for tissues. Furthermore, the best intervention time for anti-resistance agents (such as osimertinib) will be explored through quantitative and dynamic analysis of EGFR-sensitive and resistant mutations in plasma cfDNA during the EGFR TKI treatment process. The final result will be presented at the meeting of the American Society of Clinical Oncology this year.

## Conclusion

### Barriers and future research

The past decade has seen great progress in clinical research and drug development in China. Investigators have not only participated in international studies but have also conducted trials based on the characteristics of eastern populations. Moreover, a series of novel targeted agents with good efficacy have been synthetized and introduced for clinical use.

Although these significant advances have changed the treatment of lung cancer in China, there are still some shortcomings that require urgent improvement. First, the development of novel agents for the treatment of lung cancer is a highly competitive field. In the annual report for drug approval in China, 2013–2015, the most active area of drug innovation and development was in the field of antineoplastic agents, particularly those for lung cancers. Second, most novel small molecular inhibitors target EGFR, PDGFR, HER2, or VEGFR; inhibitors targeting ALK, RAF, MEK, and JAK are relatively rare. Furthermore, most of the domestic novel anticancer agents are structural modifications of agents that are already on the market or are undergoing research; innovative design is rare. The pharmaceutical industry should shift the focus of their work from generic agents to the development of pioneer agents [28].

To improve the efficacy of biomarker-driven clinical trials, innovative technologies such as liquid biopsy and NGS may be effective strategies for rapid identification and comprehensive molecular profiling of lung cancer [29]. To date, Chinese investigators have found liquid biopsy to be efficacious in monitoring the dynamic change of driver genes during treatment in several studies (FASTAC-2/NCT00883779; CTONG0901/NCT01024413) [30]. Furthermore, in a prospective phase II study (BENEFIT, NCT02282267), the EGFR mutation status was determined by cfDNA in the plasma. However, despite the enthusiasm encompassing liquid biopsy and NGS, their clinical utility, such as their sensitivity and accuracy, remains unproven. To use novel technologies in clinical trials, further efforts and studies are still needed.

## Abbreviations

ALK: Anaplastic lymphoma kinase
ALT: Alanine transaminase
AST: Aspartate transaminase
CFDA: China Food and Drug Administration
CNS: Central nervous system
DCR: Disease control rate
EGFR: Epidermal growth factor receptor
FGFR: Fibroblast growth factor receptor
HER2: Human epidermal growth factor receptor 2
HGF: Hepatocyte growth factor
IHC: Immunohistochemistry
MET: Mesenchymal–epithelial transition
NSCLC: Non-small cell lung cancer
ORR: Objective response rate
OS: Overall survival
PFS: Progression-free survival
PI: Principal investigator
RP2D: Recommended phase II dose
TKI: Tyrosine kinase inhibitor
US FDA: The United States Food and Drug Administration
VEGFR: Vascular endothelial growth factor receptor
